# Routing in Wireless Sensor Networks Using an Ant Colony Optimization (ACO) Router Chip

**DOI:** 10.3390/s90200909

**Published:** 2009-02-13

**Authors:** Selcuk Okdem, Dervis Karaboga

**Affiliations:** Erciyes University, Engineering Faculty, Computer Engineering Department, 38039, Kayseri, TR, Turkey; E-Mail: karaboga@erciyes.edu.tr

**Keywords:** Wireless sensor networks, routing, ant colony optimization

## Abstract

Wireless Sensor Networks consisting of nodes with limited power are deployed to gather useful information from the field. In WSNs it is critical to collect the information in an energy efficient manner. Ant Colony Optimization, a swarm intelligence based optimization technique, is widely used in network routing. A novel routing approach using an Ant Colony Optimization algorithm is proposed for Wireless Sensor Networks consisting of stable nodes. Illustrative examples, detailed descriptions and comparative performance test results of the proposed approach are included. The approach is also implemented to a small sized hardware component as a router chip. Simulation results show that proposed algorithm provides promising solutions allowing node designers to efficiently operate routing tasks.

## 1. Introduction

Due to advances in low-power wireless communications, low-power analog and digital electronics, the development of low-cost and low-power sensor nodes that are small in size has received increasing attention. Sensor nodes have the ability to sense the environment nearby, perform simple computations and communicate in a small region. Although their capacities are limited, combining these small sensors in large numbers provides a new technological platform, called Wireless Sensor Networks (WSNs). WSNs provide reliable operations in various application areas including environmental monitoring, health monitoring, vehicle tracking system, military surveillance and earthquake observation [1-2].

Although WSNs are used in many applications, they have several restrictions including limited energy supply and limited computation and communication abilities. These limitations should be considered when designing protocols for WSNs. Because of these considerations specific to WSNs, many routing schemes using end-to-end devices and MANETs [3] are inappropriate for WSNs.

In sensor networks, minimization of energy consumption is considered a major performance criterion to provide maximum network lifetime. When considering energy conservation, routing protocols should also be designed to achieve fault tolerance in communications. In addition, since channel bandwidth is limited, protocols should have capability of performing local collaboration to reduce bandwidth requirements [4].

The basic method to transfer information from a sensor node to the base is called flooding. In this method, information is disseminated by all the nodes as well as the base node. The broadcasting operation to all over the network consumes too much node resources such as energy and bandwidth. Heinzelman *et al.* proposed SPIN family protocols that disseminate all the information in the network assuming that all nodes are potential base nodes [5]. However SPIN's data advertisement operation does not guarantee data delivery. In this respect multi-path routing protocols promise advantages. The use of multiple paths to transfer data to the base enhances the reliability of WSNs. Directed diffusion [6] is a candidate method for multi-path routing. However, directed diffusion may not be suitable for those monitoring applications which require periodic data transfers.

The optimization of network parameters for a WSN routing process to provide maximum network lifetime might be considered as a combinatorial optimization problem. Many researchers have recently studied the collective behavior of biological species such as ants as an analogy providing a natural model for combinatorial optimization problems [7-10]. Ant colony optimization (ACO) algorithms simulating the behavior of ant colony have been successfully applied in many optimization problems such as the asymmetric traveling salesman [11], vehicle routing [12] and WSN routing [8,13,14].

Singh *et al.* [15] proposed an ant based algorithm for WSN routings. However, this algorithm does not consider the main specifics of WSN structures, including energy related issues. Zhang *et al.* [16] proposed ant based algorithms for WSNs; their study includes three routing algorithms named SC, FF, and FP. The algorithms are successful with initial pheromone settings to have a good system start-up, but the SC and FF algorithms are not quite effective in latency, while providing better energy efficiency. Besides, the FP algorithm, while providing high success rates of data delivery, consumes much higher energy than the FC and FF algorithms. The Energy Efficient Ant Based Routing Algorithm for WSNs (EEABR) [17], based on a ACO metaheuristic, is another proposed ant based algorithm to maximize the lifetime of WSNs. The algorithm uses a good strategy considering energy levels of the nodes and the lengths of the routed paths. In this paper, we have compared the performance results of our ACO approach to the results of the EEABR algorithm. Various differently sized networks are considered, and our approach gives better results than EEABR algorithm in terms of energy consumption.

The main goal of our study was to maintain network life time at a maximum, while discovering the shortest paths from the source nodes to the base node using a swarm intelligence based optimization technique called ACO. A multi-path data transfer is also accomplished to provide reliable network operations, while considering the energy levels of the nodes. We also implement our approach on a hardware component to allow designers to easily handle routing operations in WSNs. The preliminary report on this work may be seen in [18]. The rest of the paper is organized as follows. In Section 2, the proposed routing scheme using ACO is explained by an illustrated example scenario. In Section 3, performance results obtained from the simulations are given. In Section 4, implementation of the approach is presented with hardware simulation results. Finally, in Section 5 we conclude our study and give our future work plan.

## 2. Proposed WSN Routing Scheme

A WSN routing task which consists of stable or limited mobile nodes and a base station is considered as the problem. To achieve an efficient and robust routing operation, major features of typical WSNs are taken into consideration. First, failures in communication nodes are more probable in WSNs than classical networks, as nodes are often located in unattended places and they use a limited power supply. Therefore the network should not be affected by a node's failure and should be in an adaptive structure to maintain the routing operation. This is performed by sustaining different paths alive in a routing task. A node transferring data to the base sends it in divided parts (as data packages) using different paths. When a failure occurs in a path, the associated data package cannot arrive at the base. To achieve guaranteed delivery, acknowledgement signals are used. In the case of an absent acknowledgement for a data package, the source node resends that package to a different path. By performing acknowledgement-associated data transfers and sustaining different paths alive, routing becomes more robust. It is obvious that some paths in this type of network would be shorter, allowing for lower energy costs. Transmission on these paths should be more frequent to reduce the total cost of energy consumed using these paths. In other words, more data packages should be transferred along shorter paths to achieve a lower energy consumption.

Second, nodes in WSNs present stringent energy constraints. They consume much more energy when they are in communication. In our proposed approach, the energy levels of the nodes should also be considered as well as the lengths of the paths. This is performed by choosing nodes having more energy in a routing task. Thus, the average network lifetime would be increased.

Third, the bandwidth of wireless links in WSNs is limited. It is important not to involve too much information about overhead of the routing task in the communications. This is also a means of preserving more energy. We propose a new communication technique using ant agents in Section 2.1.

Fourth, some node mobility should be allowed in some specific WSN applications. In our approach, nodes are considered to be normally stable. However, probable changes in node locations do not preclude network operation safety. Instead, it causes some setup stage to organize paths well. However, transfer of data packages is still performed in this stage as quality grows over time by exploring new paths.

To summarize the operation of the routing scheme, a node having information for the base station initializes the routing task by transferring data in packages to different neighbor nodes. Each node then chooses other neighbor nodes and so on. Thus, paths towards the base are formed and each routing operation supplies some information about optimum paths for the consequent routing tasks. While performing this operation, some agents (artificial ants) are used to achieve efficient routing. This operation is explained in the following section.

### 2.1. ACO Approach

In the ACO based approach, each ant tries to find a path in the network, providing minimum cost. Ants are launched from a source node *s* and move through neighbor repeater nodes *r_i_*, and reach a final destination node (sink) *d*. Whenever, a node has data to be transferred to the destination which is described as a base or base station, launching of the ants is performed. After launching, the choice of the next node *r* is made according to a probabilistic decision rule (1):

(1) $$P_{k}(r,s)=\left\{ \begin{array}{l} \frac{{\left[\tau (r,s)\right]}^{\alpha }\cdot {\left[\eta (r,s)\right]}^{\beta }}{\underset{r\in R_{s}}{\text{∑}}{\left[\tau (r,s)\right]}^{\alpha }\cdot {\left[\eta (r,s)\right]}^{\beta }}\text{if}\;k\notin \mathrm{tab}u^{r}\\ 0\;\text{otherwise} \end{array}\right.$$

where *τ* (*r,s*) is the pheromone value, *n* (*r,s*)is the value of the heuristic related to energy, *R_s_* is the receiver nodes. For node *r, tabu^r^* is the list of identities of received data packages previously. α and β are two parameters that control the relative weight of the pheromone trail and heuristic value. Pheromone trails are connected to arcs. Each *arc*(*r,s*) has a trail value. *τ* (*r,s*) ∈ lsqb;0,1rsqb; Since the destination *d* is a stable base station, the last node of the path is the same for each ant travel. The heuristic value of the node *r* is expressed by equation (2):

(2) $$\eta (r,s)=\frac{{\left(I-e_{r}\right)}^{-1}}{\sum_{n\in R_{s}}{\left(I-e_{n}\right)}^{-1}}$$

where *I* is the initial energy, and *e_r_* is the current energy level of receiver node *r*. This enables decision making according to neighbor nodes' energy levels, meaning that if a node has a lower energy source then it has lower probability to be chosen. Nodes inform their neighbors about their energy levels when they sense any change in their energy levels.

In traditional ACO, a special memory named M*_k_* is held in the memory of an ant to retain the places visited by that ant (which represent nodes in WSNs). In equation (1), the identities of ants (as sequence numbers) that visited the node previously, are kept in the node's memories, instead of keeping node identities in ant's memories, so there is no need to carry M*_k_* lists in packets during transmission. This approach decreases the size of the data to be transmitted and saves energy. In equation (1) each receiver node decides whether to accept the upcoming packet of ant *k* or not, by checking its tabu list. So, the receiver node *r* has a choice about completing the receiving process by listening and buffering the entire packet. If the receiver node has received the packet earlier, it informs the transmitter node by issuing an ignore message, and switches itself to idle mode until a new packet arrives.

After all ants have completed their tour, each ant *k* deposits a quantity of pheromone Δ*τ^k^*(*t*) given in equation (3), where $J_{w}^{k}(t)$ is the length of tour, *w^k^* (*t*) which is done by ant *k* at iteration *t*. The amount of pheromone at each connection ((*l*(*r,s*))of the nodes is given in equation (4). In WSNs, $J_{w}^{k}(t)$ represents the total number of nodes visited by ant *k* of tour *w* at iteration *t*:

(3) $$\Delta \tau^{k}(t)=1/J_{w}^{\text{k}}(t)$$

(4) $$\tau (r,s)(t)\leftarrow \tau (r,s)(t)+\Delta \tau (r,s)(t),\forall l(r,s)\in w^{k}(t),k=1,\ldots ,m$$

Pheromone values are stored in a node's memory. Each node has information about the amount of pheromone on the paths to their neighbor nodes. After each tour, an amount of pheromone trail Δ*τ^k^* is added to the path visited by ant *k*. This amount is the same for each arc(*r, s*) visited on this path. This task is performed by sending ant *k* back to its source node from the base along the same path, while transferring an acknowledgement signal for the associated data package. Increasing pheromone amounts on the paths according to lengths of tours, *J_w_* (*t*) would continuously cause an increasing positive feedback. In order to control the operation, a negative feedback, the operation of pheromone evaporation after the tour is also accomplished in equation (5). A control coefficient *ρ* ∈ (0,1) is used to determine the weight of evaporation for each tour [19]:

(5) $$\tau_{ij}(t)\leftarrow (1-\rho )\tau_{ij}(t)$$

In simulations, ACO parameter settings are set to values 1 for *α*, 5 for *β*, and 0.5 for *ρ*, which were experimentally found to be good by Dorigo [20].

### 2.2. Protocol Operations

#### 2.2.1. Disseminating Data

Several nodes become sources having information about an event which takes place nearby. This information is disseminated towards to the base node by the help of neighbor nodes behaving as repeaters. Associated data from each source is split to *N* pieces called data parts. An integer value *N* also represents the number of ant agents involving in each routing task.

**Figure 1.**
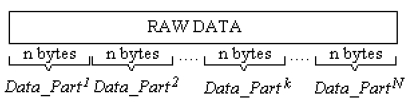
Raw data splits.

The data about an event, provided by the source node, is named raw data. Raw data contains information such as source node identification, event identification, time and data about the event. Raw data is split by the source node into *N* parts as shown in Figure 1. The size of each data part is chosen according to buffer size of the WSN communication chip.

**Figure 2.**
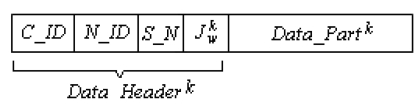
Data package content.

After splitting the raw data into parts, each part is associated with routing parameters to build a data package ready to transfer. These parameters are code identification (describing the code following as data, error or acknowledge) *C_ID*, next node identification (to which the package is transferred) *N_ID*, package number (also represents the ant agent) *k, S_N* is the sequence number, $J_{w}^{k}$ which contains the number of visited nodes so far, and the *k*th data part as given in Figure 2. In this figure, the group of the first four fields is named the data header. When delivery of all data packages is accomplished, the base combines them into raw data.

**Figure 3.**
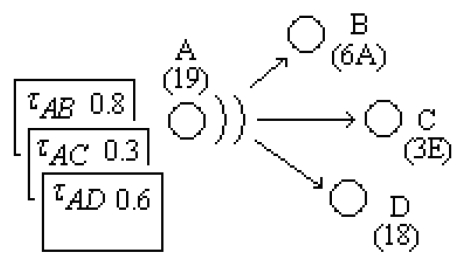
ACO parameters memorized in the nodes.

In Figure 3, a node participation in a routing for the 10th packet is given as an example. In the figure, Node A has just received a data package (whose agent number is given as ten) and makes a decision about the next destination for that package.

The next node (the next address of package tarasfer) is chosen by using equation (1) providing the highest *P^k^_ij_*. In this example, Node B (identified by 6A) more likely to be chosen as arc(*A, B*) has a higher pheromone value (*τ_AB_* =0.8). Energy levels of the neighbor nodes B, C, and D have also importance on this decision rule. If Node B is then chosen, *N_ID* field is updated as “6AHex” and the package is broadcasted. Nodes C and D also hear this broadcast. They check *N_ID* field and understand that this ID doesn't correspond to them, so they discard the package immediately after only listening to the *N_ID* field. Node B also checks *N_ID* field of the package. With the approval of ID by node B, and ensuring *S_N* isn't in the list of tabus of node B, then $J_{w}^{k}$ is updated by incrementing by one. Then, the next node is determined to update the *N_ID* field by performing the same operations as the previous node (Node A) had done. Defining the size of data package is essential, because the number of ants is equivalent to the number of packages. So, package size must be defined by the initial system settings according to the average size of event data and hardware specifics. Minimum size of data in a data package consisting of MAC frames should be as long as the size of a frame in lower level MAC. If the 802.11 MAC layer protocol is in use, it should be considered that data payload in a frame contains up to 2,312 bytes. In the simulation settings in Section 3, this size is set to 1 Mb.

#### 2.2.2. Acknowledgement Method

**Figure 4.**
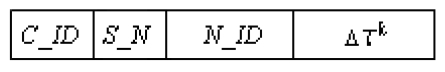
Acknowledgement signal content.

**Figure 5.**
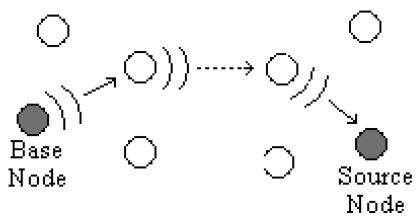
Transmission an acknowledgement signal.

After the dissemination operation by the source and neighbor nodes is done, the base gets data in parts from several paths. The main idea of using different paths is to provide reliable transfer operations in case of breakdown of the major routes. To prevent package loss in these paths, acknowledgement signals are used. After receiving a package, the base decides the pheromone value to be added on that route by evaluating $J_{w}^{k}$ in equation (3). Then, it forms an acknowledgement signal as in Figure 4 and broadcasts it towards to the source, using the same path which has been used for the associated package. This operation is illustrated in Figure 5.

A node receiving an acknowledgement signal first checks the *S_N* value. If the number is found in the node memory (tabu list) that has been inserted previously while disseminating associated packages, then this signal is broadcasted to other neighbors along the path. The node also updates the pheromone value by adding Δ*τ^k^* specified in the signal by using equation (4). If the node ID is not found in the memory then this signal is discarded and no further operation is done. Thus, Δ*τ^k^* is added along the routed path. The source node waits for the acknowledgement signal of each data package. In case of an absent acknowledgement signal, caused by errors in the path, that package should be disseminated again.

#### 2.2.3. Informing Energy Levels of Neighbor Nodes

Energy levels of neighbor nodes are required for decision rule (1), so each node needs to report its energy level to its neighbors. When a change is sensed in the energy level, reporting is carried out by broadcasting. The change is more likely after an active participation of listening or transmitting. So, they could sense their energy status immediately after any participation.

## 3. Simulations and Results

In this section, we present the performance results of the simulation experiments. To accomplish the experiments, a parallel discrete event-based platform was developed in MATLAB. In the simulation, a free space radio propagation model is used. In order to verify the success of the proposed approach, an energy-efficient ant-based routing algorithm EEABR [17], a well known ACO based WSN routing algorithm, was used to make comparisons. The simulation is capable of running packet level experiments, and uses parameter values of a hardware named MICAz mote [21], specifications of which are given in Table 1. Experimental results are obtained for the two algorithms which are the one proposed in the approach described in Section 2 and the EEABR algorithm in [17]. The ACO parameters and the specifics of hardware are set to the values specified in Section 2.1 and Table 1.

**Figure 6.**
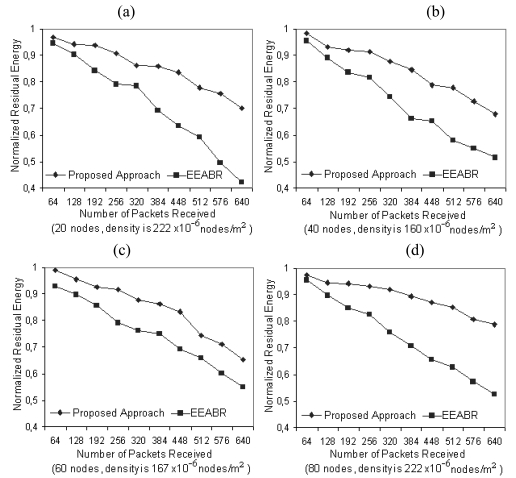
Average residual energy for different WSNs having various number of nodes.

The results of the proposed approach and EEABR algorithm were obtained using the same settings for the simulation. Deployment of sensors are made by random distribution over 200 × 200 m^2^ (10 nodes), 300 × 300 m^2^ (20 nodes), 400 × 400 m^2^ (30 nodes), 500 × 500 m^2^ (40 nodes) and 600 × 600 m^2^ when 50, 60, 70, 80 and 100 nodes are used to monitor a static phenomenon. The location of the phenomenon and the sink node are not known. In Figure 6, network densities are calculated by dividing the number of nodes by the total area. In the scenario, the nodes near the phenomenon send the relevant data through the neighbor nodes to the sink by consuming energy. As the number of packets received by the sink increases, the average residual energy of the nodes (that is used as the metric) decreases as shown in Figure 6. In the simulations, the proposed approach gives better results, providing more network lifetime and consuming less energy, particularly for the networks having high densities.

**Figure 7.**
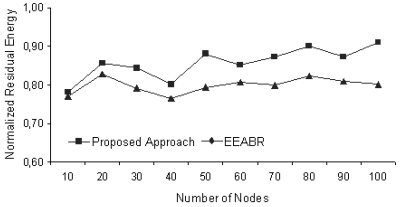
Average residual energy after 256 packets are received.

Figure 7 shows normalized average residual energy after 256 packets are received by the sink node for different WSNs having various number of nodes. From the figure, it is seen that the difference in energy levels increases as the number of nodes in the network grows as much as up to 10 percent.

## 4. Implementation of ACO Algorithm

**Figure 8.**
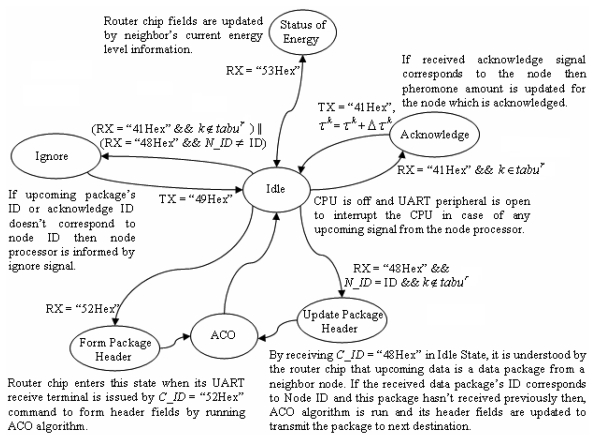
State Diagram of Communication Protocol.

Because of space constraints on the sensor nodes, designers should consider the space requirements of the hardware components they use. A small sized chip having the routing capability mentioned so far offers advantageous solutions for the design stage. By using a separate chip, the full computational power of the main processor could be used for sensor node tasks (e.g. sensing, data processing) specific to itself instead of allocating some of that power to relatively complex routing tasks. Moreover, it is not required that node designers code routing protocols which need detailed knowledge of the issue. Instead, a hardware component ready to perform dynamic routing operations is proposed. Routing tasks are performed under the guidance of the router chip embedded a novel routing protocol using ACO as designers have no need to know any routing considerations. The chip uses a simple communication protocol illustrated in detail in Figure 8. In Table 2, the main specifications of this router chip are given.

The proposed ACO routing protocol is embedded on a programmable device which has only four pins, requiring minimum connections and smaller space of only 30 mm^2^ of SOIC footprint, suitable for tiny node designs. The router chip has two pins for RX and TX of the UART terminal (9,600 baud rate) used to communicate with the master processor of the node. The circuit using the proposed router chip was tested in the Proteus simulation platform [22] and the performance results given in Table 3 were obtained.

As mentioned in Section 2.2, protocol operations are described in three groups which are disseminating data, acknowledging and informing of energy levels of neighbor nodes. All of these tasks are performed under the guidance of the router chip as the main processor of the node communicates with this chip. The chip has a ROM to hold the ACO algorithm, a processor to run the algorithm, and RAM memory space to hold the ACO parameters. The signals (used for communication to router chip) and their descriptions are given in Table 4.

## 5. Conclusions

In this paper we have presented a new protocol for WSN routing operations. The protocol is achieved by using an ACO algorithm to optimize routing paths, providing an effective multi-path data transmission method to achieve reliable communications in the case of node faults. We aimed to maintain network life time at a maximum, while data transmission is achieved efficiently, so an adaptive approach is developed according to this goal. The proposed approach is compared to a well-known ant based algorithm named EEABR using an event-based simulator. The results show that our approach offers significant reductions of energy consumption which is used as a performance metric for different sized WSNs. We also implemented our approach on a small sized hardware component requiring minimum connections suitable for tiny node designs and we developed an easy method for handling the routing tasks by using the proposed router chip. We also tested the ACO algorithm running on the router chip and obtained its performance results, including response times of the chip. Response time of the header request for the routing operation would be satisfactory for many WSNs where transmission speed is not essential. The proposed ACO approach for WSN routings and its hardware implementation seem to be a promising solution for node designers. As future work, it is planned to improve our routing approach to be effective in proper WSN settings, including nodes having high mobility. The improved approach will also be studied in network types that include multiple sink nodes.

## Figures and Tables

**Table 1.** Parameter values of the hardware used in simulations.

**Parameter Name**	**Value**
Frequency	2,400 MHz
Transmit(TX) data rate	250 Kb/s
RF power	-10 dBm
Receive (RX) Sensivity	-94 dBm
Current Draw in Transmit Mode	11 mA
Current Draw in Receive Mode	19.7 mA
Battery	2 × (1,250 mAH, 1.5V)
Packet Size	1 Mb

**Table 2.** Specifications of the router chip.

**Parameter Name**	**Value**
Power Up	64ms
Initial Configuration	139μs
Neighbor Update	7.5ms
CPU Speed	5 MIPS
Operating Temperature	-40°C to 125°C
Operating Voltage	2V to 5.5V
Pin Count	8
Package Type	Soic-8

**Table 3.** Response times of the router chip.

**State**	**Response Time**
Form Package Header (by running ACO)	25 ms
Update Package Header (by running ACO)	25 ms
Acknowledge (From the Base)	625 μs
Acknowledge (From the Others)	<5 μs
Ignore	140 μs

**Table 4.** Signals used in router chip communication.

**Signals**	**Fields in the Signal**	**Descriptions of the Fields(respectively)**
**Form Package Header**	*C_ID(52Hex)*	Command ID
**Update Package Header**	*C_ID(48Hex), N_ID, k*, and	Command ID, Node ID, Agent Number and Number of Visited Nodes
**Ignore**	*C_ID(49Hex)*	Command ID
**Acknowledge**	*C_ID(41Hex), k, N_ID*, and Δ*τ^k^*	Command ID, Agent Number, Node ID and Value of Agent Trail
**Status of Energy**	*C_ID(53Hex), N_ID*, and *E_LE*	Command ID, Node ID, and Energy Level
